# Understanding the Psychological Impact of Lockdown: Combining Quantitative and Qualitative Analysis of Emergency Presentations

**DOI:** 10.1192/bjo.2022.339

**Published:** 2022-06-20

**Authors:** Tom Scurr

**Affiliations:** Derriford Hospital, Plymouth, United Kingdom

## Abstract

**Aims:**

The pandemic of the COVID-19 variant caused a near-global lockdown, and the psychological impact of the direct effects of the virus, along with the resulting lockdown periods cannot be overestimated. Referrals made to the Liaison Psychiatry service at Derriford hospital during the 2020 lockdown were audited to better understand effects on patients’ mental health and resulting emergency presentations to services. These data were then used to identify areas for improvement, in order to tailor services to better support the population during recovery from the current lockdown, and for planning for future similar events.

**Methods:**

Referrals to the Derriford Liaison Psychiatry service between the 1st and 12th of May 202 were audited, totalling 106 referrals and a subsequent 87 assessments. Quantitative data on patient demographics, presentation, and outcomes was extracted from assessments along with qualitative data on patients’ subjective experiences from the initial lockdown period for thematic analysis. Routine data were used for comparator time periods from 2019, and during the second 2021 lockdown.

**Results:**

Despite a lower number of presentations to ED during the first lockdown, the data demonstrate a higher acuity in presentations with more referrals for admission under section. The lockdown is shown to have particularly affected those with pre-existing psychiatric and physical comorbidity, along with specific patient groups. Thematic analysis confirms this, showing the diverse factors contributing to emergency presentations and demonstrating the increased stress of life in the home under lockdown. Comparisons between the qualitative and quantitative data confirm that patient experiences directly match both the routinely collected data and prior research. The project also revealed a reliance on private and third sector organisations for signposting on from assessments, and highlighted frequent changes to services during lockdown as a source of confusion for both patients and staff.

**Conclusion:**

The scale of impact identified affirms that exploration of the lockdown's contribution to presentation should be routine, particularly for identified at-risk patient groups. Areas frequently highlighted by patients can be used to fully explore the impact of lockdown on presentation during assessment. Patient information for self-referral needs to be regularly updated given frequent changes in service provision. Staff also need to be kept up to date on changing service structure at handover meetings.

